# Chimeric Peptidomimetic Antibiotic Efficiently Neutralizes Lipopolysaccharides (LPS) and Bacteria-Induced Activation of RAW Macrophages

**DOI:** 10.1021/acsinfecdis.2c00518

**Published:** 2023-02-15

**Authors:** Ali Javed, Cornelis J. Slingerland, Thomas M. Wood, Nathaniel I. Martin, Femke Broere, Markus H. Weingarth, Edwin J. A. Veldhuizen

**Affiliations:** † Faculty of Veterinary Medicine, Department of Biomolecular Health Sciences, Division Infectious Diseases & Immunology, Section Immunology, Utrecht University, 3584 CL Utrecht, The Netherlands; ‡ Biological Chemistry Group, Institute of Biology Leiden, Leiden University, 2333 BE Leiden, The Netherlands; § NMR Spectroscopy, Bijvoet Centre for Biomolecular Research, Department of Chemistry, Faculty of Science, Utrecht University, 3584 CS Utrecht, The Netherlands

**Keywords:** peptides, innate immunity, sepsis, LPS neutralization, immunomodulation

## Abstract

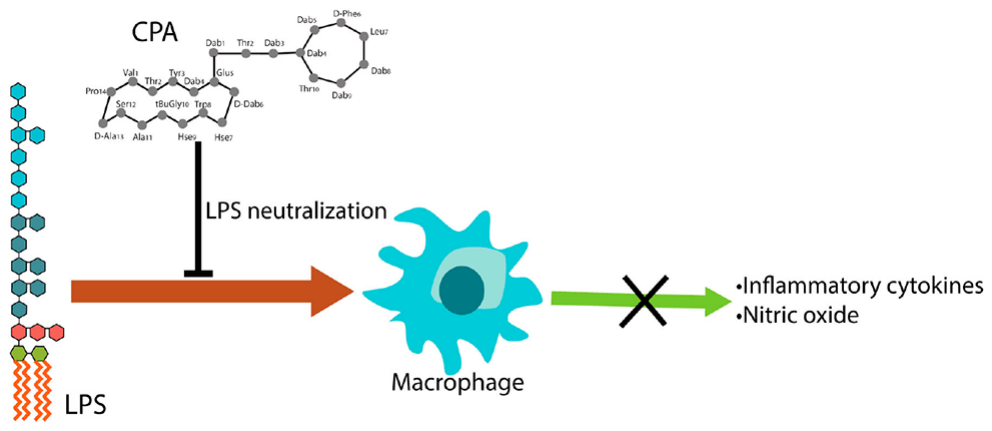

Peptide antibiotics have gathered attention given the urgent need to discover antimicrobials with new mechanisms of action. Their extended role as immunomodulators makes them interesting candidates for the development of compounds with dual mode of action. The objective of this study was to test the anti-inflammatory capacity of a recently reported chimeric peptidomimetic antibiotic (CPA) composed of polymyxin B nonapeptide (PMBN) and a macrocyclic β-hairpin motif (MHM). We investigated the potential of CPA to inhibit lipopolysaccharide (LPS)-induced activation of RAW264.7 macrophages. In addition, we elucidated which structural motif was responsible for this activity by testing CPA, its building blocks, and their parent compounds separately. CPA showed excellent LPS neutralizing activity for both smooth and rough LPSs. At nanomolar concentrations, CPA completely inhibited LPS-induced nitric oxide, TNF-α, and IL-10 secretion. Murepavadin, MHM, and PMBN were incapable of neutralizing LPS in this assay, while PMB was less active compared to CPA. Isothermal titration calorimetry showed strong binding between the CPA and LPS with similar binding characteristics also found for the other compounds, indicating that binding does not necessarily correlate with neutralization of LPS. Finally, we showed that CPA-killed bacteria caused significantly less macrophage activation than bacteria killed with gentamicin, heat, or any of the other compounds. This indicates that the combined killing activity and LPS neutralization of CPA can prevent unwanted inflammation, which could be a major advantage over conventional antibiotics. Our data suggests that immunomodulatory activity can further strengthen the therapeutic potential of peptide antibiotics and should be included in the characterization of novel compounds.

The global increase in antimicrobial resistance (AMR) requires the development of new antimicrobial agents that use new mechanisms of actions.^1−3^ Drug discovery approaches to tackle antimicrobial resistance involves, for example, harnessing natural products of so-called unculturable bacteria or the development of synthetic compounds based on natural product scaffolds.^4−8^ Interestingly, among the new antibiotic lead structures with new targets described recently, many belong to macrocyclic peptides.^9^

In the pursuit of new antimicrobials, antimicrobial peptides (AMPs) are considered to be promising candidates due to their structural and functional variety.^10,11^ Naturally occurring AMPs are short cationic amphiphilic peptides produced by a diverse range of organisms from eukaryotes to prokaryotes as a vital part of their host defense mechanism against microbial pathogens. Besides direct antimicrobial activity, a large array of immunomodulatory activities have been assigned to AMPs, which is why they are often referred to as host defense peptides, a term that better reflects the entirety of their functions.^12,13^ Based on these properties, AMPs are also explored for their immunomodulatory potential.^9^ Approaches to synthesize structural and functional mimics of AMPs have also been pursued to develop novel peptide antibiotics with optimized activity.^9^

Sepsis is the most common cause of death among critically ill patients in noncardiac intensive care units.^14^ It is characterized by a strong systemic inflammatory response resulting from the excessive stimulation of innate immune cells by molecules from microbial pathogens, particularly lipopolysaccharides (LPSs).^15^ Immune cells recognize LPS by the pattern recognition receptor toll-like receptor 4 (TLR4). Modulation of TLR signaling could therefore play a crucial role in controlling sepsis. Eukaryotic AMPs, mainly cathelicidins and defensins, have been reported to modulate the immune system via LPS neutralization, leukocyte recruitment, chemokine expression, and macrophage differentiation.^16,17^ In the case of microbial AMPs, several immunomodulatory activities have also been described when they are used in mammalian in vitro or in vivo systems. For example polymyxin B (PMB) (Figure 1A) has LPS binding capacity and has been reported to suppress immune cell activation in response to bacterial endotoxins by inhibiting proinflammatory cytokine expression.^18,19^ Interestingly, PMB and LL-32 (a variant of the human cathelicidin LL-37) have been shown to neutralize LPS induced inflammation in vivo and in vitro, not only by direct interaction but also by affecting the receptor signaling pathways.^20^ Another microbial AMP, Nisin-A has also been reported to regulate apoptosis and T cell proliferation, in addition to LPS neutralization.^18^ Polymyxin B nonapeptide (PMBN) (Figure 1B), a cyclic peptide obtained from enzymatic processing of PMB, is capable of binding to LPS, rendering Gram-negative bacteria susceptible to antimicrobials^21^ and has also been shown to reduce LPS-triggered inflammatory markers,^22^ suggesting a similar immunomodulatory potential as PMB. These studies suggest a potential dual role for natural and synthetic peptides as direct killing and immunomodulatory therapeutic agents in cases of infections and sepsis.

**Figure 1:**
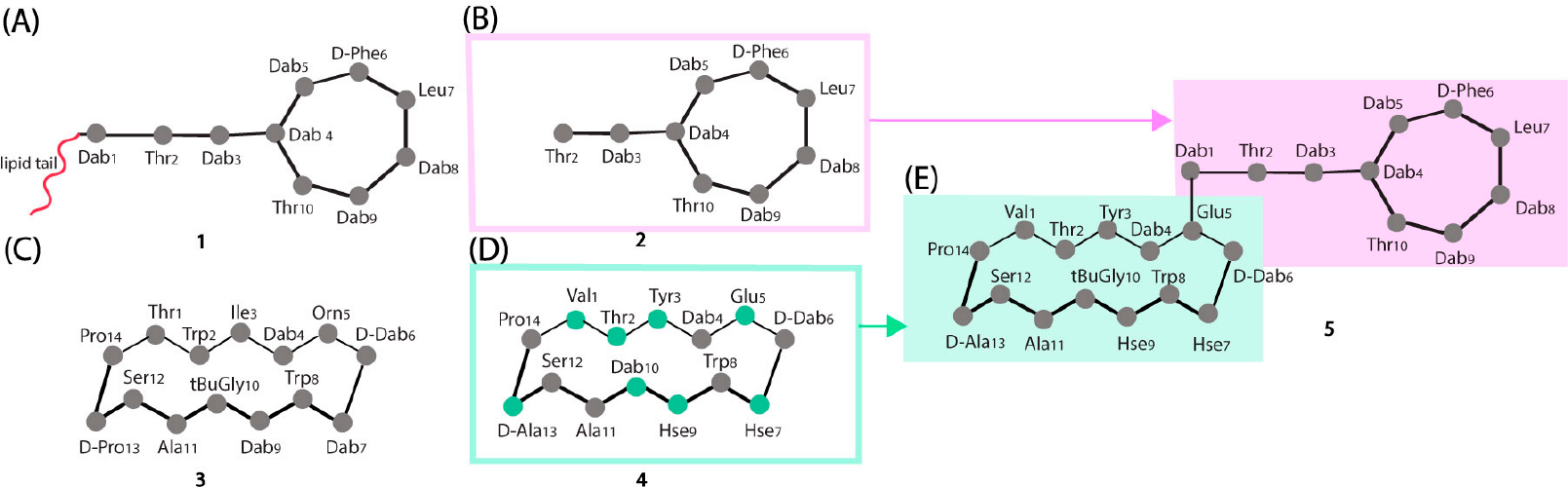
Schematic structures of the five peptides included in the study: (A) structure of polymyxin B; (B) structure of polymyxin B nonapeptide; (C) structure of murepavadin; (D) structure of macrocyclic β-hairpin motif (amino acid residues different from the parent compound showed in green color); and (E) structure of the recently reported chimeric peptidomimetic antibiotic consisting of β-hairpin mimetic ligated to the polymyxin B nonapeptide.

The development of synthetic peptides that bind to LPS and/or β-barrel outer membrane proteins (OMPs) is a promising approach to develop peptide antibiotics that target the outer membrane (OM) of Gram-negative pathogens. One such synthetic peptide is murepavadin (Figure 1C), which is a β-hairpin mimic of the cationic AMP Protegrin-1, and it is among the most notable OMP-targeting antibiotics.^23^ Murepavadin is currently in clinical development by the pharmaceutical company *Spexis* to treat lung infections caused by *Pseudomonas* (*P*.) *aeruginosa*.^24^ It is a narrow-spectrum antibiotic with selective activity against *P. aeruginosa*,^23^ which it kills by targeting the OMP LptD.^23,25^ Potential anti-inflammatory or immunomodulatory activities have not been reported thus far for murepavadin. Recently, researchers at Polyphor AG developed a chimeric peptidomimetic antibiotic (CPA) consisting of a murepavadin-inspired macrocyclic β-hairpin motif (MHM) covalently linked to PMBN (see Figure 1D,E).^26^ Polyphor’s CPA was constructed based on the hypothesis that the activity of the β-hairpin macrocycle would synergize with the LPS binding capacity of PMB. They subsequently showed that such CPA constructs exhibit enhanced antibacterial activity against ESKAPE pathogens.^26^ Interestingly, in addition to binding LPSs, CPAs were shown to target the OMP BamA, the most vital component of the β-barrel assembly machine (BAM) that plays a key role in OMP folding.^27^ Importantly, CPA also retained potent antibacterial activity in vivo.^26^

While polyphor’s CPA was designed to kill Gram-negative bacteria, we hypothesized that the molecules on which this conjugate is based on (murepavadin-derived β-hairpin macrocycle and PMBN) could also make CPA a good candidate to stimulate anti-inflammatory and immunomodulatory effects. The purpose of this study was to test CPA for its anti-inflammatory activity in terms of suppressing the LPS and bacteria stimulated immune cell activation and to systematically compare it to the activity of its separate building blocks (PMBN and MHM) and their parent compounds PMB and murepavadin in its antibacterial and anti-inflammatory efficacy.

## Results

### Antibacterial Activity

**Figure 2:**
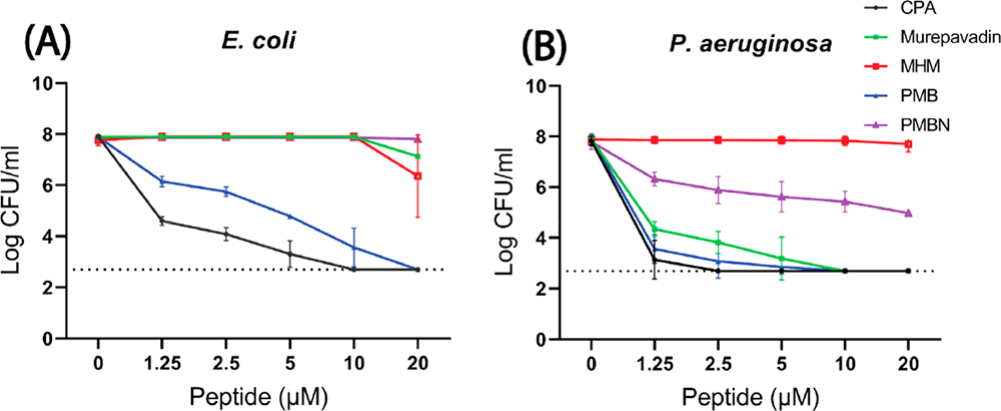
Determination of MBC values for (A) *E. coli* and (B) *P. aeruginosa*. Surviving bacterial colonies were detected after incubation with tested peptides for 3 h in MHB. Shown are mean ± SD of three independent experiments; dashed line shows the detection limit of the assay.

Colony count assays were used to determine which of the tested peptides had bactericidal activity against *Escherichia* (*E*.) *coli* and *P. aeruginosa* (Figure 2A). CPA was most active against *E. coli*. Increasing concentrations of the peptide resulted in decreasing numbers of viable bacteria, with already a 2-log decrease (from the starting density of 10^6^ CFU/mL) at 1.25 μM to complete killing at 10 μM. PMB showed slightly lower activity but still reached an MBC value between 10 and 20 μM. In contrast, PMBN, MHM, and murepavadin were unable to affect the viability of *E. coli* up to 20 μM in these assays. In the case of *P. aeruginosa* (Figure 2B), the most active peptide again was CPA, which completely killed all bacteria at 2.5 μM, followed by PMB and murepavadin, which had MBC values between 5 and 10 μM, respectively. PMBN and MHM were unable to significantly decrease the number of viable bacteria up to 20 μM.

### LPS Neutralization

**Figure 3:**
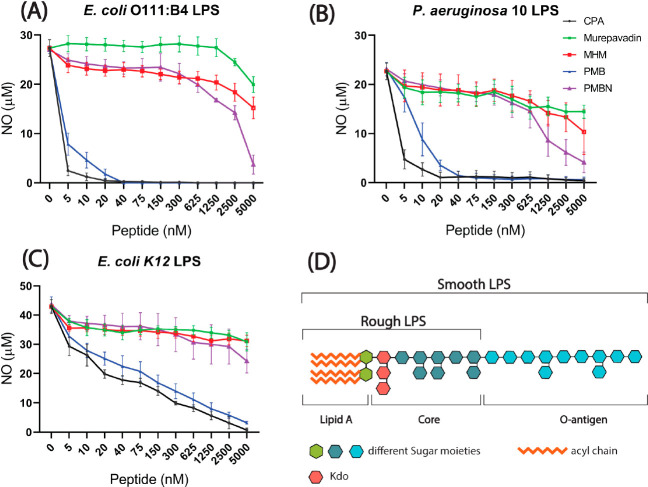
Nitric oxide inhibition in LPS stimulated RAW264.7 by peptide antibiotics. RAW264.7 cells were stimulated with smooth type LPSs (A) *E. coli* O111:B4 LPS and (B) *P. aeruginosa* LPS and rough type LPS (C) *E. coli* K-12 LPS in the presence of different concentrations of peptides: CPA, murepavadin, MHM, PMB, and PMBN. NO production was measured by Griess assay. Shown are the mean ± SEM of three independent experiments. (D) Schematic diagram of the general structure of lipopolysaccharides. It consists of three main subunits: lipid A, the core region, and the O-antigen. Lipid A and the core-region form rough (R)-type LPS. Lipid A, the core-region, and the O-antigen together form smooth (S)-type LPS.

LPSs can activate macrophages by binding to TLR4 and trigger downstream signaling, resulting in nitric oxide production and release of cytokines. Three different LPS variants were tested: a rough type LPS (from *E. coli* K-12) and two smooth type LPSs (from *E. coli* O111:B4 and *P. aeruginosa* 10). Irrespective of the LPS source, CPA and PMB were dose-dependently active in inhibiting LPS-induced nitric oxide production of RAW264.7 macrophages, while murepavadin and MHM were not (Figure 3). PMBN, in case of both types of smooth LPS, partially inhibited nitric oxide production only at 5 μM, the highest concentration tested. CPA was most active in neutralizing all three types of LPS. It completely neutralized *E. coli* O111:B4 LPS and almost completely neutralized *P. aeruginosa* 10 LPS at a concentration of 20 nM, while *E. coli* K-12 LPS was neutralized at 5 μM. PMB was slightly less active than CPA. These results indicate that the building blocks of CPA alone do not have the efficient LPS neutralizing activity as the chimera possesses itself. Furthermore, all peptides seem to be more active in neutralizing *E. coli* O111:B4 LPS and *P. aeruginosa* 10 LPS than *E. coli* K-12 LPS.

**Figure 4:**
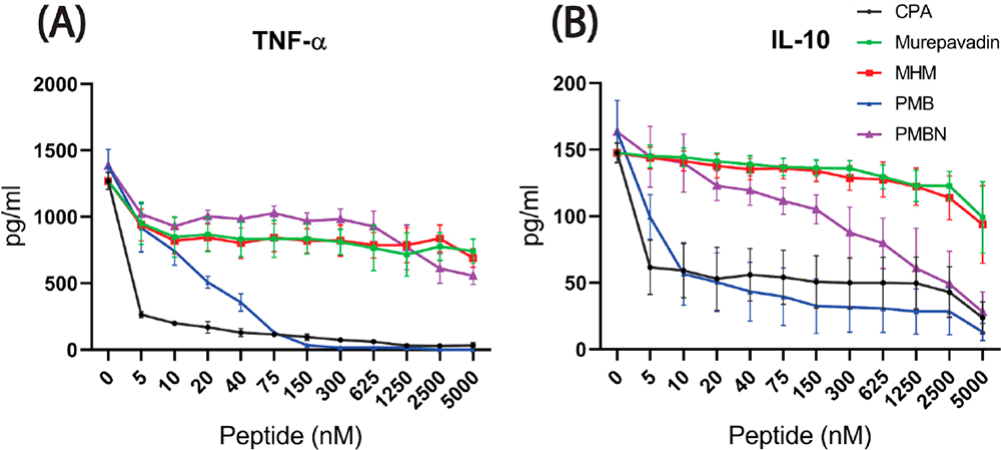
Peptides modulation of cytokine expression in RAW264.7 cells stimulated with LPS. RAW macrophages were stimulated with 20 ng/mL *E. coli* LPS in the presence of different concentrations of peptide antibiotics. (A) TNF-α and (B) IL-10 release was measured by ELISA in duplicates. Shown are the mean ± SEM of three independent experiments.

The capacity of peptides to modulate cytokine release in LPS (*E. coli* O111:B4) stimulated RAW macrophages was determined using ELISA to measure TNF-α and IL-10 levels in the supernatant of the macrophages. These results showed the same trend in LPS neutralization capacity as seen for nitric oxide production. CPA sharply decreased the TNF-α and IL-10 release by RAW264.7 macrophages at a concentration of 5 nM and then showed dose-dependent inhibition of cytokine release (Figure 4). PMB showed slightly less activity compared to CPA. Murepavadin and MHM were unable to inhibit the release of both types of cytokines up to a concentration of 5 μM, while PMBN was found to inhibit IL-10 release dose-dependently but at concentrations much higher than those required for CPA or PMB.

### Cytotoxicity

**Figure 5:**
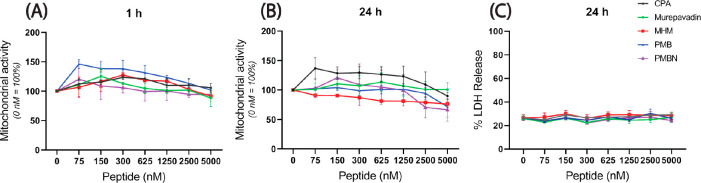
Cytotoxic effects of the peptides against RAW264.7 cells. Cells incubated with the peptides during 1 or 24 h were tested using a (A and B) WST-1 assay, indicating cell metabolic activity and (C) LDH release assay indicating cell membrane permeability. Shown are the mean ± SEM of three independent experiments.

To rule out the possibility of cytotoxicity of the peptides as the reason for inhibition of nitric oxide and cytokine release, the cytotoxic effect of these peptides on RAW 264.7 cells was tested by incubating the cells for 1 or 24 h with the peptides up to the concentration as used in LPS neutralization assays. These studies showed that none of the peptides reduced the cell viability or increased the LDH release at either time point or either concentration (Figure 5).

### LPS Binding

**Figure 6:**
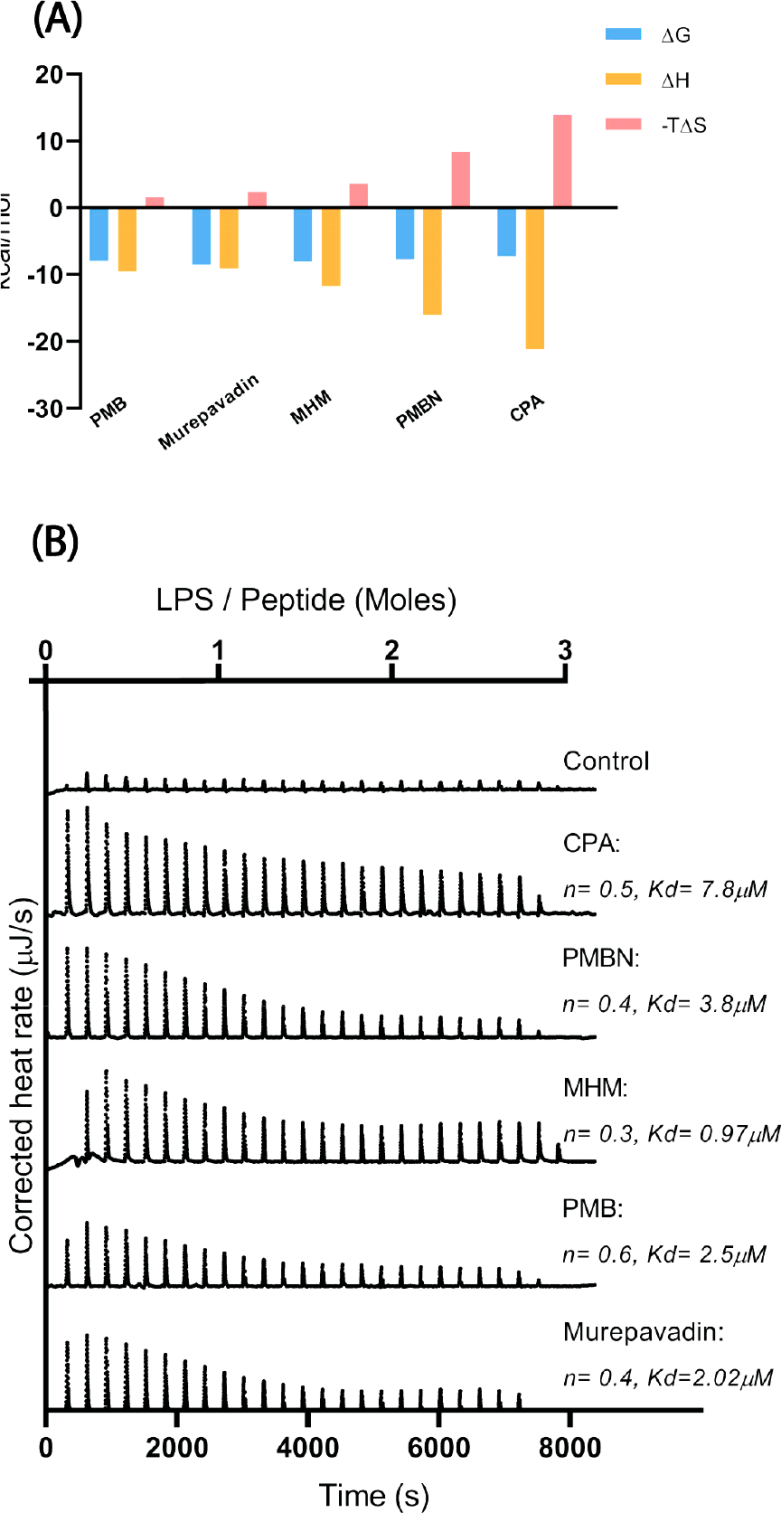
(A) Binding profile of LPS and the peptides using ITC. Binding signatures (Δ*G*, Δ*H*. and −TΔ*S*) plotted for binding events of LPS with peptides. (B) Spectra of the interaction between LPS and peptides. Murepavadin, PMB, MHM, PMBN, and CPA (30 μM) were titrated against 200 μM *E. coli* O111:B4 LPS, and heat rates were recorded. Shown is a representative of two measurements.

Next, we used isothermal titration calorimetry (ITC) to investigate if the peptides directly bound to LPS, as this could account for the neutralization of LPS-induced macrophage activation. ITC experiments showed exothermic binding to LPS for all peptides, as indicated by the negative enthalpy (Δ*H*) values (Figure 6). With *K*_d_ values in the μM range for all peptides, no marked differences were observed for peptide–LPS binding (Figure 6). Strikingly, murepavadin and MHM, i.e., the two peptides that were completely non-active in neutralization of LPS-induced macrophage activation (Figure 3), also showed binding to LPS comparable to the other peptides. All the peptides showed enthalpy-driven binding with unfavorable entropy as indicated by the entropy factor (−TΔ*S*), suggesting that ionic interactions were at the basis of the interaction of peptides with LPS.

### Silent Killing

**Figure 7:**
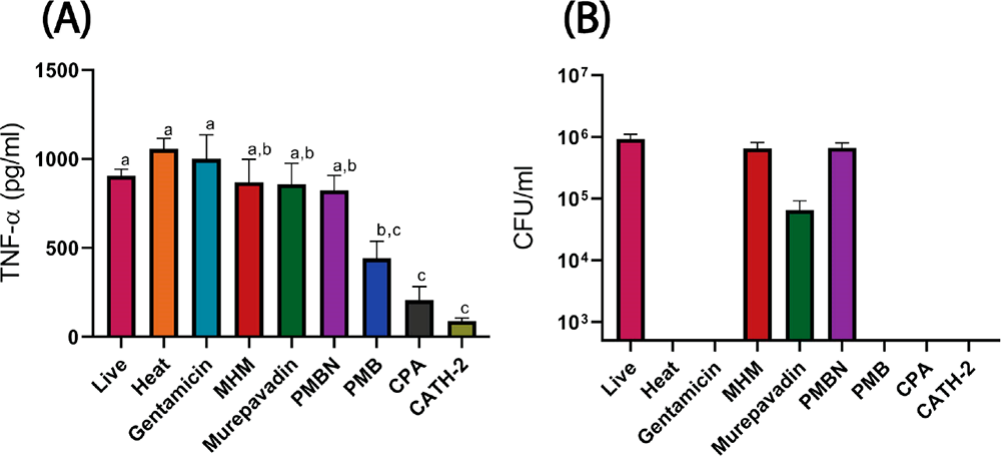
(A) Determination of macrophage activation in response to viable and nonviable bacteria. *P. aeruginosa* was treated with gentamicin, heat treatment, CATH-2 (control), CPA, murepavadin, MHM, PMB, or PMBN (20 μM of each) and subsequently added to RAW264.7 macrophages. Production of TNF-α was measured using ELISA after 2 h of treatment. (B) Viability of *P. aeruginosa* after treatment was checked by plating on TSA plates. Data is plotted as the mean ± SEM of three independent experiments. Significant differences were determined by ordinary one-way ANOVA (Tukey’s multiple comparison test). Data points that are significantly different from each other are denoted by different letters (*p* < 0.05).

So-called silent killing experiments were performed to determine if nonviable bacteria, killed with peptides, still trigger an inflammatory response by macrophages. RAW264.7 macrophages were stimulated with *P. aeruginosa* treated with the peptides, or as controls with Gentamicin-killed, heat-killed, or viable untreated bacteria. After 2 h incubation of RAW cells with (treated) bacteria, TNF-α levels were measured by ELISA as a read out for macrophage activation. These experiments showed that heat-killed and Gentamicin-killed bacteria actually stimulated macrophage activation to almost same level as viable untreated bacteria. Similarly, when bacteria were killed with murepavadin or MHM, no significant reduction in macrophage activation was observed compared to viable bacteria. However, when CPA and chicken cathelicidin 2 CATH-2 (for which silent killing was originally described^29^) were used to kill bacteria, a significantly reduced immune response was observed. CPA was very efficient in suppressing immune activation after killing as the immune activation of macrophages, in terms of TNF-α secretion, was significantly lower (*p* < 0.05) than for the other peptides in the study (Figure 7). PMB-killed bacteria also showed a tendency towards significantly lower (*p* < 0.05) macrophage stimulation. These data again show a gain of function for the bicyclic chimera compared to its building blocks and parent compounds.

## Discussion

In this study, it was confirmed that CPA had strong antibacterial activity against *E. coli* and *P. aeruginosa*, with higher activity than its parent compounds, murepavadin (for *P. aeruginosa*) and PMB, while the CPA fragments MHM and PMBN were themselves inactive. Overall, these observations correlate well with the described activity of these compounds.^24,26,30−32^ The lack of the acyl chain of PMBN compared to the parent molecule PMB (Figure 1) most likely causes this reduced antibacterial activity, and it could be hypothesized that in CPA this addition of MHM replaced the acyl-chain, thereby regaining antimicrobial activity. However, a second explanation could be that the CPA conjugate possesses a more varied and efficient mechanism of action compared to PMB. The finding that CPA targets BamA to initiate its antibacterial activity favors this hypothesis.^26^

The effect of the peptides on the neutralization of smooth type LPS variants induced macrophage stimulation yielded some very interesting insights. Again, MHM and PMBN had little-to-no activity compared to CPA, showing the requirement of both building blocks together to neutralize LPS. This is in itself very interesting because the construct of CPA was discovered using antimicrobial killing as a read-out parameter not LPS neutralization. Considering that in these neutralization experiments PMB is also very active, the same line of thought can be followed about CPA’s activity, which is that replacing the acyl chain of PMB with the MHM-block could possibly restore or even slightly increase the LPS neutralization activity. However, the fact that CPA showed even higher LPS neutralization than PMB is highly intriguing and indicates that it should not be considered as just a ‘slightly adjusted PMB molecule’. The increased neutralizing activity of CPA could indicate a different bacterial target compared to its parent compounds as seen for antibacterial specificity.^23,26^ The efficiency of CPA in neutralizing smooth type LPSs was greater than that of the other compounds tested. For rough type LPS (*E. coli* K-12), (Figure 3C), similar trends in neutralizing activity were seen, although at somewhat higher concentrations than in case of smooth type LPSs (Figure 3A,B). This might provide some insights on the LPS–peptide interaction sites, suggesting that the specific LPS structure plays a role in the neutralization mechanism.

There could be multiple mechanisms of inhibition of LPS mediated macrophage activation. One mechanism is the direct binding of peptides to LPS thereby blocking interaction of LPS with TLR4. ITC was performed to determine the thermodynamic parameters governing the peptide–LPS interactions. All peptides bound to LPS with comparable binding affinity in the micromolar range, under the experimental conditions used. As shown by the entropy factor (−*T*Δ*S*) and binding enthalpy (Δ*H*) values (Figure 6), the binding interactions of all peptides are enthalpy driven (exothermic). This indicates an important role for electrostatic interactions between the peptides and negatively charged LPS. Our results are also in line with a previous study that reports the biophysical interaction of PMB and PMBN with LPS as a strong exothermic reaction.^33^ The binding affinity of polycationic peptides for LPS relies on different factors like peptide–LPS charge ratio, fluidization of the acyl chains of the LPS, and the reaggregation of LPS and, thus, can vary with different peptides and different LPS species.^33,34^

Given that murepavadin and MHM bound to LPS in ITC studies but did not neutralize LPS in terms of inhibiting nitric oxide and cytokine release of macrophages (Figure 3), our data suggest that direct binding of LPS is not sufficient for neutralization. This can be explained in multiple ways. First, binding studies using ITC are performed in simple buffer systems because of the sensitivity of the method. It is possible that in cell culture conditions peptide–LPS interactions are different. Another important aspect is that high concentrations of LPS and peptides are required in ITC studies to be able to measure the heat produced by binding. This higher concentration means that LPS will be in an aggregated micellar structure in ITC studies while it will be in a monomeric form in the macrophage activation studies that require low LPS concentrations. Possibly this concentration-dependent structural change of LPS molecules in solution can explain the lack of correlation between binding and neutralizing LPS. Interestingly, endotoxins are described to be more active in a specific aggregated form.^35,36^ LPS activation of immune cells occurs as the LPS aggregates bind to the host’s membrane molecules such as LBP, which then monomerize LPS molecules and transfers to CD14, which further facilitates its binding to TLR4/MD2 complex.^37^ A model of LPS neutralization suggests that LPS neutralizing peptides bind and reaggregate the LPS in a way so that the lipid A part of LPS is organized in a multilamellar structure,^20,38,39^ thus affecting the overall LPS organization. This altered structure is unable to activate immune cells. Finally, a possibility unrelated to the physical characteristics of LPS is that some peptides can affect the host cells directly, thereby rendering them less susceptible to LPS activation. Some cathelicidins and PMB were able to inhibit LPS induced cell activation by interacting with and modifying the organization of surface receptors, thus affecting intracellular signaling pathways.^20^ Further studies unraveling at what point CPA blocks TLR4 activation would be very interesting to further elucidate the peptides’ potential.

Neutralization of purified LPS is an important indication of potential immune modulatory activity of antimicrobial peptides, but the effect of peptides on neutralizing the immune activation potential of whole bacteria is much more relevant. Therefore, the peptides were assessed for their neutralization capacity in an in vitro model where RAW macrophages were exposed to whole bacteria (viable and nonviable). Results showed that only CPA was able to significantly neutralize macrophage activation (Figure 7) by (non-) viable bacterial treatment. This activity of CPA was comparable to CATH-2 for which this dual activity of killing bacteria and reducing subsequent excessive inflammation has been described and was related to its LPS neutralizing activity.^29^ This characteristic of CPA provides it with a major advantage over normal antibiotics especially toward treatment of infections that could potentially lead to sepsis. The dual activity, or “silent killing”, observed for CPA (and CATH-2) is actually not that common; some AMPs are mostly effective killers while other AMPs like for example LL-37 are not very active in killing bacteria in physiological conditions but are very efficient in neutralizing the LPS of nonviable bacteria.^40^ This implicates that potential synergy would also be possible in vivo between the chimeric peptidomimetic antibiotics and the hosts own arsenal of immune molecules, which would even further strengthen the potential for therapeutic use. These aspects need to be further explored in future studies.

## Conclusions

In conclusion, our study reveals the additional capacity of CPA to suppress LPS-induced immune cell activation and in doing so kills bacteria without the immune activation often seen with other antibacterial compounds. Future studies on the mechanism of LPS neutralization and other immunomodulatory aspects of CPA may provide further insights into the potential for developing optimized peptide antibiotics with multiple modes of action as a means of improving therapies for the treatment of sepsis.

## Methods

### Peptides

Murepavadin was synthesized by solid phase synthesis (SPPS) as follows: CTC resin was functionalized with Fmoc-Pro, yielding a resin loading of 0.4 mmol/g. Peptide synthesis was performed on a 0.1 mmol scale. After deprotection (20% piperidine in DMF), Fmoc-d-Pro was installed by a double coupling (4 eq BOP, 8 eq DIPEA; 1 h, followed by overnight coupling). Following an end-capping acetylation step with acetic anhydride (0.5 mL, combined with 0.8 mL of DIPEA and DMF to a total volume of 5 mL), Fmoc-Ser, and Fmoc-Ala were subsequently coupled by manual SPPS (resin bound AA/Fmoc-AA/BOP/DIPEA, 1:4:4:8 mol equiv) with couplings in DMF for 1 h and deprotections with 20% (v/v) piperidine/DMF for 5 min, followed by 25 min. Following deprotections, the resin was washed by DCM (3 × 8 mL) and DMF (3 × 8 mL). The tetrapeptide loaded resin was then transferred to a CEM Liberty Blue microwave peptide synthesizer, and the synthesis was completed using the same reagents as indicated above with couplings (4 min) at 50 °C and deprotections (1 min) at 90 °C. Amino acid side chains were protected as follows: tBu for Ser and Thr and Boc for Dab, Orn, and Trp.

After completion of the linear peptide and final Fmoc removal, the resin was treated with 20% HFIP/DCM (v/v) for 1 h, followed by a second HFIP/DCM treatment for 15 min. The cleavage solutions containing the crude (side-chain protected) peptide were combined and concentrated and coevaporated with DCM, and the peptide was cyclized overnight by treatment with HATU (3 equiv), HOAt (3 equiv), and DIPEA (6 equiv) in DMF (80 mL). The mixture was then concentrated, and the peptide was deprotected by treatment with TFA/TIPS/H_2_O (95/2.5/2.5 (v/v/v), 8 mL) for 1.25 h. The crude peptide was precipitated in ice-cold MTBE (2 × 40 mL), and the pellet was washed with MTBE, after which it was freeze-dried from a *t*-BuOH/H_2_O mixture.

Pure murepavadin (≥95%) was obtained after reverse-phase HPLC purification on a preparative HPLC system (BESTA-Technik), equipped with a ECOM Flash UV detector monitoring at 214 nm. A C18 column (25 mm × 250 mm, 10 μm, Dr. Maisch) was employed. The following solvent system was used at a flow rate of 12 mL/min: solvent A, 0.1% TFA in water/acetonitrile (95/5); solvent B, 0.1% TFA in water/acetonitrile (5/95). Gradient elution was as follows: 95:5 (A/B) for 3 min, 95:5 to 60:40 (A/B) over 47 min, 60:40 to 100:0 (A/B) over 3 min 0:100 (A/B) for 4 min, and then reversion back to 95:5 (A/B) over 1 min, 95:5 (A/B) for 2 min.

Peptide purity was confirmed by analytical HPLC, using a Shimadzu Priominence LC-2030 system with a C18 column (Shimadzu, 3.0 mm × 150 mm, 3 μm) at 30 °C, with UV monitoring at 214 nm. The following solvent system, at a flow rate of 0.5 mL/min, was used: solvent A, 0.1% TFA in water/acetonitrile (95/5); solvent B, 0.1% TFA in water/acetonitrile (5/95). Gradient elution was as follows: 95:5 (A/B) for 2 min, 95:5 to 60:40 (A/B) over 23 min, 60:40 to 100:0 (A/B) over 1 min 0:100 (A/B) for 2 min, and then reversion back to 95:5 (A/B) over 1 min, 95:5 (A/B) for 2 min.

The peptides CPA and MHM were prepared using Fmoc solid phase peptide synthesis (SPPS) as described before.^24^ CATH-2 was synthesized by Fmoc-chemistry at China Peptides (CPC scientific, Sunnyvale, CA). Stock solutions of peptides (2.5 mM CPA and 1.6 mM other peptides) were prepared in distilled water (or for CPA in 2% DMSO, 0.002% tween-20) and further diluted in appropriate medium as indicated in those assays. A schematic representation of the tested peptides in this study is depicted in Figure 1.

### Bacterial Strains

Bacterial strains used in this study were clinical isolates of *Escherichia coli* and *Pseudomonas aeruginosa* provided by Utrecht University Medical Center, Department of Medical Microbiology, Utrecht, The Netherlands. Both strains were cultured in Mueller Hinton broth (MHB) (Millipore, Sigma-Aldrich).

### Cell Culturing

RAW264.7 cells (ATCC TIB-71) were cultured in Dulbecco’s modified eagle medium (DMEM) (Gibco, Thermo Fisher Scientific, Waltham, MA) supplemented with 10% fetal calf serum (FCS) (Bodinco B.V., Alkmaar, The Netherlands) and 100 units/mL penicillin and 100 μg/mL streptomycin at 37 °C, 5.0% CO_2_.

### Colony Count Assay

Logarithmic growth phase of an o/n bacterial culture was obtained by diluting it 1:100 in MHB and then incubating it for 3 h at 37 °C while shaking. After that, the OD_620_ of the bacterial suspension was measured and the bacterial solution was diluted to 2 × 10^6^ colony forming units/mL (CFU/mL). Bacteria and peptides in MHB were mixed 1:1 (v/v) and incubated for 3 h at 37 °C in a polypropylene round-bottom 96 well-plate (Corning Costar, Glendale, AZ). Subsequently, 10-fold dilutions were made and plated out on Tryptone Soy Agar (TSA) plates and incubated for 24 h at 37 °C to count colonies.

### Nitric Oxide Inhibition Assay

RAW264.7 cells were seeded (5 × 10^4^ cells/well) in a 96-well plate and left at 37 °C for overnight adherence. Then, cells were stimulated with four types of LPS: 20 ng/mL LPS originating from *E. coli* O111:B4 (LPS-EB, InvivoGen), 50 ng/mL LPS from *P. aeruginosa* (PA-10 LPS, Sigma -Aldrich), and 5 ng/mL LPS from *E. coli* K-12 (LPS-EK, InvivoGen) in the presence of 0–5 μM peptides in DMEM for 24 h. After incubation, the nitrite content (corresponding to nitric oxide produced but quickly oxidized in watery solutions to nitrite) in the supernatant was measured using the Griess assay.^28^

### Enzyme-Linked Immuno Sorbent Assay (ELISA)

A sandwich ELISA was used to measure TNF-α and IL-10 concentrations in the supernatant of the same treatment (24 h) used for nitrite measurement, using ELISA Duoset kits (R&D systems, Minneapolis, MN) according to the manufacturer’s protocol.

### Cell Viability Assay

RAW264.7 cells (5 × 10^4^ cells/well) were seeded as described above. Cells were stimulated with different concentrations of peptides in DMEM for 1 or 24 h. After incubation, the medium was replaced by 100 μL of culture medium containing 10% water-soluble tetrazolium 1 (WST-1) (Roche, Basel, Switzerland). Colorimetric changes were measured, after 15–20 min of incubation, at 450 nm using a FLUOstar Omega microplate reader (BMG Labtech GmbH, Ortenberg, Germany). Cell viability was calculated as percentage viability with the no peptide control set to 100% viability.

### Lactate Dehydrogenase (LDH) Assay

RAW264.7 cells were stimulated with the peptides (0–5 μM in DMEM) as described above. After 24 h, cytotoxicity was measured as fraction of lactate dehydrogenase (LDH) release in the supernatant compared to maximum LDH release of unstimulated cells treated with Triton X-100 detergent to completely lyse cells using the Cyto Tox 96 nonradioactive cytotoxicity kit (Promega), according to the manufacturer’s instructions.

### Isothermal Titration Calorimetry (ITC)

ITC measurements were performed in a low volume NanoITC (TA Instruments-Waters LLC, New Castle, DE). Two hundred micromolar LPS (from *E. coli* O111:B4) was prepared in 50% PBS. Peptides of 30 μM concentration were prepared in identical buffer. Three hundred microliters of the peptide solution was added to the chamber (chamber volume is 169 μL, so the rest of the volume flows out and rests around the chamber), and 50 μL of LPS was added to the syringe. At 37 °C, 2 μL of LPS solution was injected into the chamber every 300 s, except the first injection, which consists of 0.96 μL. The experiments were performed at 37 °C while stirring at 300 rpm. The data was analyzed with the NanoAnalyze Software (TA Instruments, Asse, Belgium).

### Simulation of Macrophages with Nonviable Bacteria

RAW264.7 cells were seeded (7.5 × 10^4^ cells/well) as described above. *P. aeruginosa* (diluted to 1 × 10^6^ CFU/mL in DMEM) was treated separately with heat (1 h at 90 °C), 1 mg/mL gentamicin (1 h at 37 °C), 20 μM CPA (1 h at 37 °C), 20 μM PMB (1 h at 37 °C), 20 μM murepavadin (1 h at 37 °C), 20 μM MHM (1 h at 37 °C), 20 μM CATH-2 (1 h at 37 °C), and DMEM only (untreated sample) (1 h at 4 °C). After treatment, the bacteria were added to the RAW264.7 cells for 2 h of incubation at 37 °C. After that, TNF-α production was determined in the supernatant by ELISA.

### Statistical Analysis

Graphpad Prism version 9.3.1471 was used for statistical analysis. Ordinary one-way ANOVA (Tukey’s multiple comparison test) was applied to test results for significant differences (*p* < 0/05).
